# Low Expression of a Circular Transcript of the Apoptosis Regulator Gene *BOK* Is Associated with Unfavorable Prognosis in Breast Cancer

**DOI:** 10.3390/biomedicines14051118

**Published:** 2026-05-15

**Authors:** Vaia K. Stafyla, Spyridon Christodoulou, Nikolaos Michalopoulos, Efthimios Poulios, Panagiotis Kokoropoulos, Christos K. Kontos, Nikolaos Arkadopoulos

**Affiliations:** 1 Fourth Department of Surgery, University General Hospital “Attikon”, National and Kapodistrian University of Athens, 12462 Athens, Greece; vaniastafyla07@gmail.com (V.K.S.); spyridon.christodoulou@yahoo.gr (S.C.); efthimios.poulios@gmail.com (E.P.); kokoropoulos@yahoo.gr (P.K.); narkado@hotmail.com (N.A.); 2 First Department of Propaedeutic Surgery, “Hippokration” General Hospital of Athens, National and Kapodistrian University of Athens, 11527 Athens, Greece; nmichal@med.uoa.gr; 3 Department of Biochemistry and Molecular Biology, Faculty of Biology, National and Kapodistrian University of Athens, Panepistimiopolis, 15771 Athens, Greece

**Keywords:** breast malignancy, circular RNA (circRNA), non-coding RNA, cancer biomarker, prognostic biomarker, tumor recurrence

## Abstract

**Background**: We recently identified multiple alternative circRNAs generated through alternative circularization of primary transcripts of the BCL2-related ovarian killer (*BOK*) gene. In the present study, we evaluated the prognostic potential of a recently discovered *BOK* circRNA, namely circ-BOK-6, in breast cancer (BC). **Methods**: Tumor specimens from a cohort of 172 female BC patients were analyzed, including paired adjacent non-cancerous breast tissue from 44 cases. circ-BOK-6 expression levels were quantified using an in-house–developed quantitative real-time PCR assay, followed by comprehensive biostatistical and survival analyses. **Results**: circ-BOK-6 expression differed between matched tumor and normal breast tissues. However, no significant associations were observed between circ-BOK-6 expression and BC clinicopathological characteristics. Importantly, after splitting at the median value, low circ-BOK-6 expression was associated with poorer disease-free survival (DFS) and overall survival (OS). Multivariate Cox regression analysis further demonstrated that the prognostic value of low circ-BOK-6 expression was independent of established prognostic factors included in the models, including the prognostic stage or the molecular subtype and the anatomic stage of the disease. Additionally, stratified analyses based on these key prognostic parameters revealed that low circ-BOK-6 expression retains prognostic significance within specific patient subgroups. **Conclusions**: These findings suggest that low circ-BOK-6 expression represents a promising independent biomarker of adverse prognosis in BC.

## 1. Introduction

Breast cancer (BC) remains the leading cause of cancer-related mortality among women worldwide, with the World Health Organization estimating approximately 2.5 million new cases and 720,000 deaths annually. The global burden of the disease is projected to rise substantially, with both incidence and mortality rates expected to increase by nearly 20% by 2035. Effective clinical management of BC depends largely on early detection, for which digital mammography continues to serve as the gold-standard screening approach [1]. Prognosis is strongly stage-dependent: the five-year survival rate exceeds 95% for patients with localized disease but drops sharply to around 33% in cases of distant metastasis. Invasive breast carcinoma of no special type represents the most prevalent histological subtype, accounting for up to 80% of cases; however, it encompasses a biologically heterogeneous group of tumors with diverse morphological and clinical characteristics [2].

Anatomic staging based on the Tumor, Node, Metastasis (TNM) system provides essential prognostic information; nevertheless, recent updates to clinical guidelines have incorporated key molecular features, including estrogen receptor (ER) and progesterone receptor (PR) status, human epidermal growth factor receptor 2 (HER2) expression, the Ki-67 proliferation index, and gene expression profiling, to establish a more precise, parallel prognostic framework [3]. Exploiting these biomarkers, malignant breast tumors are stratified into four principal molecular subtypes. Luminal A, the most prevalent subtype, is characterized by high hormone receptor expression and low proliferative activity, which is associated with a favorable prognosis and strong responsiveness to endocrine therapy. In contrast, luminal B tumors exhibit lower hormone receptor levels, increased proliferation, and may display *HER2* co-amplification, contributing to reduced endocrine sensitivity and a more aggressive clinical course with a higher risk of recurrence [4]. Tumors with HER2 overexpression, driven by amplification of the *HER2* gene, were historically associated with poor outcomes; however, the advent of targeted therapies has remarkably improved patients’ prognosis [5]. Finally, triple-negative BC (TNBC), defined by the absence of ER, PR, and HER2 expression, is frequently linked to *BRCA1* and/or *BRCA2* mutations and early disease onset, and is characterized by an aggressive phenotype with high rates of recurrence, metastasis, and poor long-term survival [6].

Circular RNAs (circRNAs) constitute a distinct class of RNA molecules characterized by a covalently closed loop structure generated through back-splicing of precursor mRNAs. They are widely expressed across diverse tissues and cell types, displaying dynamic and context-dependent expression patterns. Although their functional roles are still being progressively defined, circRNAs have been implicated in the regulation of gene expression through multiple mechanisms. These include acting as microRNA (miRNA) sponges, interacting with RNA-binding proteins (RBPs), and modulating transcription via direct interactions with RNA polymerase II or transcription factors. Additionally, a subset of circRNAs has been reported to possess coding potential and give rise to functional peptides [7]. Dysregulated circRNA expression has been extensively documented in human cancers, including BC [8,9,10]. In this context, circRNAs may function either as oncogenic drivers or tumor suppressors, contributing to key processes such as apoptosis, metabolic reprogramming, therapeutic resistance, invasion, and metastasis [11,12]. Their notable stability in bodily fluids, combined with their functional relevance, underscores the promising potential of circRNAs as minimally invasive biomarkers for disease monitoring and risk stratification [7].

BOK (BCL2-related ovarian killer) is a relatively understudied member of the BCL2 protein family [13]. The designation “ovarian killer” originates from its initial identification in ovarian tissue; however, subsequent studies have demonstrated its expression across multiple tissue types, with particularly high levels observed in reproductive organs [14,15]. Although BOK is generally considered to exert pro-apoptotic activity, the presence of a BCL2-homology 4 (BH4) domain—typically associated with anti-apoptotic proteins—introduces ambiguity regarding its precise functional role [16]. Evidence from ovarian cancer research supports a pro-apoptotic function for BOK that appears to operate independently of BAX and BAK1, while also indicating that it may modulate cellular responses to chemotherapeutic agents in ovarian carcinoma [17]. In addition, alternative splicing of *BOK* gives rise to transcript variants that may reflect its functional diversity in cancer. Post-transcriptional and/or translational regulation of BOK was shown to be able to determine BC cell survival and death [18]. Moreover, downregulated BOK expression enhances epithelial–mesenchymal transition and cell migration via the Wnt signaling pathway in BC cells [19]. Nevertheless, to date, no studies have investigated the potential of *BOK* to generate circRNAs under either physiological or pathological conditions.

Alternative splicing of *BOK* pre-mRNA molecules gives rise to a diverse repertoire of circRNAs, several of which have previously been identified in established ovarian and prostate human cell lines by members of our group [20]. Despite their widespread presence and potential involvement in regulating host gene mRNA expression during tumorigenesis, the functional roles and the potential clinical relevance of these circRNAs remain largely uncharacterized. Among them, circ-BOK-6, a *BOK* circRNA originally discovered in ES-2, an established fibroblast-like cell line isolated from the ovary of a patient with clear cell carcinoma, is also expressed in BC. circ-BOK-6 has been predicted to bind miR-4267 and possess three N^6^-methyladenosine (m^6^A) sites [20]. Therefore, the present study investigates, for the first time, the clinical significance and biomarker potential of circ-BOK-6 in BC.

## 2. Materials and Methods

### 2.1. MCF-7 Cell Line Propagation

The human BC cell line MCF-7 was obtained from the American Tissue Culture Collection (ATCC) and propagated in Dulbecco’s Modified Eagle Medium (Biosera, Cholet, France) containing 2 g/L glucose, 10% FBS, and 1% penicillin/streptomycin, in a humidified incubator at 5% CO_2_, 95% humidity, and 37 °C.

### 2.2. Patients and Tissue Collection

In this study, malignant tumor samples from 172 non-consecutive female patients diagnosed with primary BC and subjected to surgery at the Fourth Department of Surgery, University General Hospital “Attikon”, Athens, Greece, were used. Moreover, in 44 of these cases, paired normal breast tissue was also available. A detailed database with biological and clinicopathological data was built; this database included the age of patients, the dimensions of the resected tumor, the infiltration of regional lymph nodes, the presence of distant metastasis, the histological and molecular subtypes, the histological grade of the tumor, the expression status of PR, ER, HER2, and mitotic rate based on the Ki-67 index. All malignant breast tumors were independently characterized by two pathologists. The anatomic (TNM) and prognostic stages were determined and recorded in the database. All breast tissue specimens were snap-frozen immediately after tumor resection.

This research study was conducted according to the guidelines of the Declaration of Helsinki and approved by the institutional Ethics Committee of the University General Hospital “Attikon”, Athens, Greece (approval number: 30; date of approval: 13 February 2013). All patients provided written informed consent.

### 2.3. Total RNA Extraction and Reverse Transcription (RT)

The fresh frozen breast tissue samples were pulverized, and then total RNA was isolated using NucleoZOL (Takara Bio Inc., Shica, Japan). Concentration measurement and quality control of the total RNA extracts were performed with a NanoDrop™ 2000 Spectrophotometer (Thermo Fisher Scientific Inc., Carlsbad, CA, USA), before RNA integrity control using agarose gel electrophoresis. Next, reverse transcription (RT) into first-strand cDNA was performed using Maxima™ Reverse Transcriptase (Thermo Fisher Scientific Inc., Carlsbad, CA, USA) and random hexamers, following the manufacturer’s protocol for first-strand cDNA synthesis, starting from 2 µg of total RNA. All RT reactions were performed in a Veriti™ Thermal Cycler (Thermo Fisher Scientific Inc.).

### 2.4. Real-Time Quantitative PCR (qPCR)

A real-time quantitative PCR (qPCR) assay was applied for the relative quantification of circ-BOK-6 against *GAPDH* mRNA, used as an endogenous reference. Furthermore, MCF-7 cDNA was used as a calibrator sample to allow the application of the comparative Ct (2^−∆∆Ct^) method for the calculations [21]. Thus, the relative expression of circ-BOK-6 in each sample was determined in RQUs by calculating the ratio of its levels to *GAPDH* transcripts, divided by the same ratio calculated for the calibrator sample.

Real-time qPCR assay optimization included designing specific divergent primers for circ-BOK-6 and convergent primers for *GAPDH* amplification [22], and optimization of the primer pair concentration. Each reaction was performed in duplicate to ensure the reproducibility of the obtained data. qPCR was performed in a QuantStudio 5 Real-Time PCR System (Thermo Fisher Scientific Inc., Carlsbad, CA, USA), using the KAPA SYBR^®^ FAST qPCR Master Mix (2X) Kit (Kapa Biosystems Inc., Cape Town, South Africa). The sequences of circ-BOK-6 primers were the following: 5′-GCCCAGAACACCTGCTCTT-3′ and 5′-TGCAGGGAGATGTGCAGCT-3′; their amplicon length was 101 bp. Importantly, circ-BOK-6 primers were divergent, so as to avoid undesirable amplicons resulting from linear transcripts, as divergent primers amplify only cDNAs produced from circRNAs during RT. Moreover, the forward primer was designed to span the back-splice junction of circ-BOK-6, to ensure the lack of by-products. Melt curve analysis confirmed the circRNA-specific amplification in each qPCR plate well.

### 2.5. Biostatistical Analysis

Comprehensive biostatistical analyses were performed using IBM SPSS Statistics (version 30, IBM Corp., Armonk, NY, USA). The distributions of circ-BOK-6 expression levels in both cancerous and normal breast tissue specimens were non-Gaussian; therefore, only non-parametric tests (the Mann–Whitney *U* test and Jonckheere–Terpstra test, where appropriate) were used to assess any potential significance of differences in circ-BOK-6 expression observed among patients’ subgroups, stratified according to distinct clinicopathological parameters. Moreover, the Wilcoxon signed-rank test was applied to assess the significance of differences in circ-BOK-6 expression between the 44 pairs of cancerous and adjacent non-cancerous breast tissues. Receiver operating characteristic (ROC) curve analysis was also performed to evaluate the ability of circ-BOK-6 expression to distinguish cancerous from normal breast tissue.

Next, the expression levels of circ-BOK-6 were split at the median of the distribution in BC tissues, and patients were hence categorized into either of two groups: circ-BOK-6–positive or circ-BOK-6–negative expression status. Tumor recurrence was defined as the endpoint for disease-free survival (DFS), whereas death attributable to BC was defined as an event in the overall survival (OS) analysis.

Kaplan–Meier survival analysis was employed to evaluate the association between circ-BOK-6 expression and patient outcomes in terms of DFS and OS. Differences between survival curves were assessed using the Mantel–Cox (log-rank) test. To evaluate the independent prognostic impact of circ-BOK-6, we utilized a Cox proportional hazards framework. The initial univariate analysis screened for significant predictors—including anatomic stage, molecular subtype, and prognostic stage of the disease—using a *p* < 0.10 entry threshold for subsequent multivariate inclusion. To prevent the confounding effects of multicollinearity, we intentionally separated anatomic staging and molecular subtyping from the integrated prognostic stage within the multivariate models, as these variables rely on shared clinical data. Moreover, bootstrap (1000 random samples) univariate and multivariable Cox regression models were built; the bias-corrected and accelerated (BCa) 95% confidence interval (CI) of each hazard ratio (HR) was also calculated. Next, stratified Kaplan–Meier survival analyses were performed in BC patients’ subgroups, as patients were stratified according to specific clinicopathological features.

Statistical significance was defined as *p* < 0.050 for all biostatistical analyses.

## 3. Results

### 3.1. Biological and Clinicopathological Characteristics of Malignant Breast Tumors

In this study, a cohort of 172 malignant breast tumors from female patients diagnosed with primary BC was used; In 44 cases, paired non-cancerous breast tissue was also available. The median age of BC patients at the time of diagnosis was 59 years (range: 32–90 years). The histological grade was assessed according to the World Health Organization (WHO) classification system; seven tumors were characterized as grade I (well-differentiated), 113 with grade II (moderately differentiated), and 52 with grade III malignant tumors (poorly differentiated). Additionally, based on the TNM stage classification, 42 malignant breast tumors were designated as stage I (24.4%), 106 as stage II (61.6%), and 24 as stage III (14.0%). The breast tumor staging and molecular subtype results are summarized in Table 1, whereas other detailed biological characteristics of the malignant breast tumors are presented in Table S1.

### 3.2. circ-BOK-6 Discriminates Between Cancerous and Normal Breast Tissues

The quantification of circ-BOK-6 expression levels in tissue specimens showed a range of 0.11 to 691.05 RQU, with a median of 14.82 RQU, in the 172 malignant tumors; in the 44 non-cancerous breast tissues that were available, the levels ranged between 0.84 and 205.71 RQU, with a median of 20.57 RQU (Table 2).

**Figure 1:**
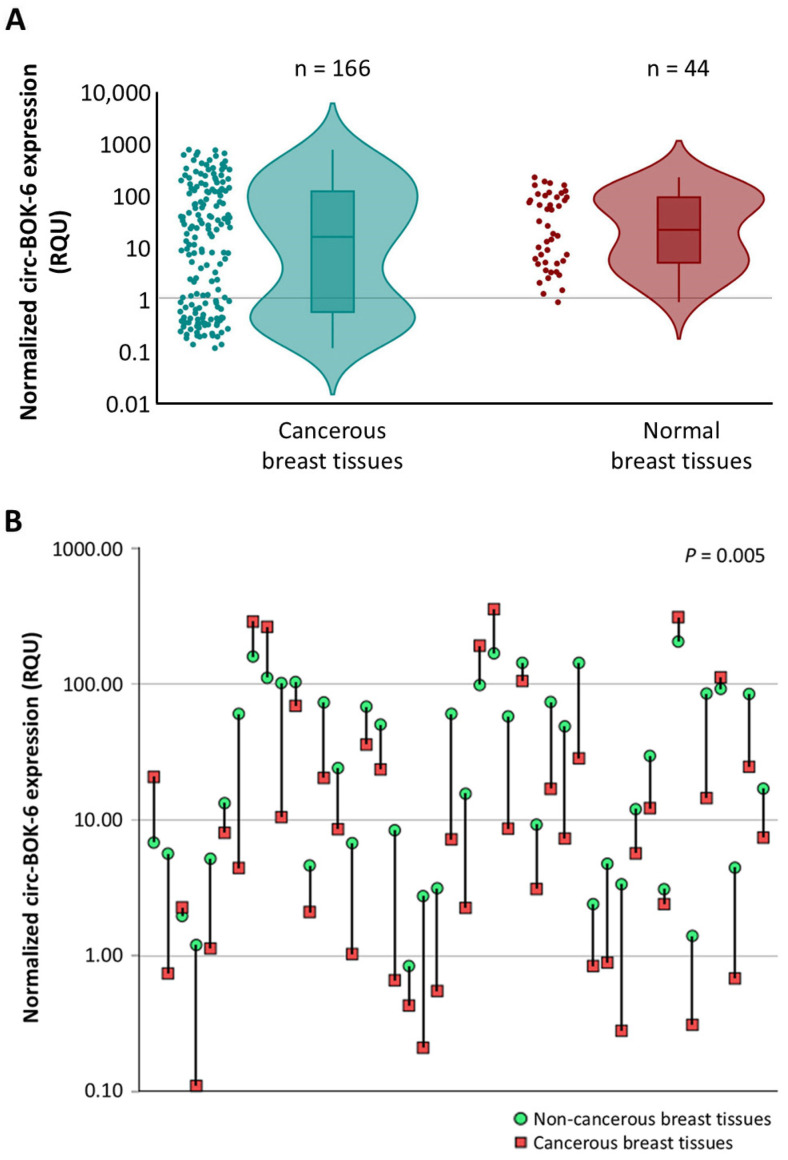
circ-BOK-6 expression in BC and normal breast tissues. (**A**) Violin plots showing the distributions of circ-BOK-6 expression levels in the two cohorts of samples. (**B**) Comparison of circ-BOK-6 expression in 44 pairs of cancerous and normal breast tissues. The *p*-value was calculated by using the Wilcoxon signed-rank test. In both plots, the y-axes are on a log scale due to the very high variability of intracellular circ-BOK-6 expression. Abbreviations: RQU, relative quantification units.

An overlap of the two distributions has been observed, thus resulting in a non-significant difference in circ-BOX-6 expression between the two cohorts (*p* = 0.17) (Figure 1A). Nevertheless, comparison of circ-BOX-6 expression levels between the 44 pairs of cancerous and normal breast tissue specimens clearly revealed significantly lower expression (*p* = 0.005) in BC (36 out of 44 tissue pairs; 81.8%) (Figure 1B).

**Figure 2:**
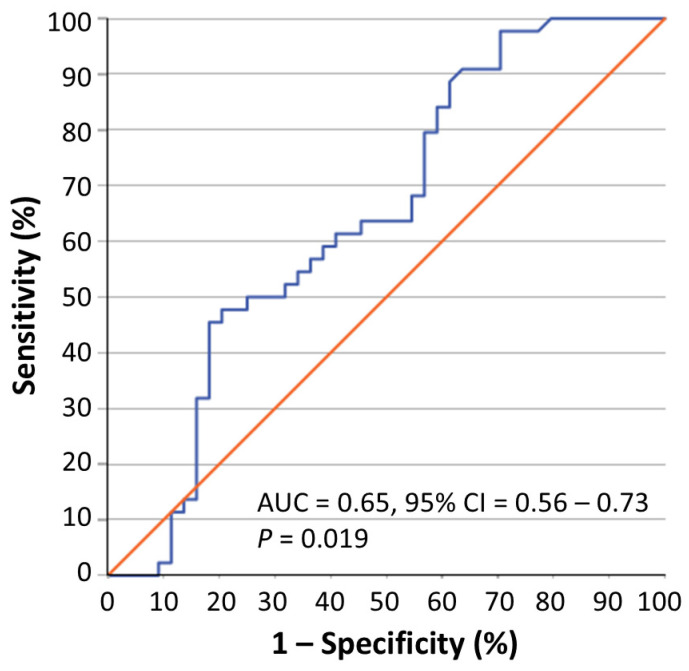
A receiver operating characteristic (ROC) curve illustrating the ability of circ-BOK-6 expression to efficiently distinguish BC from paired normal breast tissue. The *p*-value was calculated by using the Mann–Whitney *U* test. Abbreviations: AUC, area under the ROC curve; CI, confidence interval.

Next, we performed ROC curve analysis, which included the 44 cancerous samples and their respective non-malignant counterparts. This analysis showed that circ-BOK-6 expression may marginally distinguish BC from paired normal breast tissue (AUC = 0.65, 95% CI = 0.53–0.76, *p* = 0.019) (Figure 2). Therefore, the diagnostic utility of circ-BOK-6 expression in BC is rather limited.

On the other hand, circ-BOK-6 expression did not differ significantly among tumors of different molecular subtype, histological grade, anatomic stage, or prognostic stage, as shown by using the Kruskal–Wallis *H* test and the Jonckheere–Terpstra test, where appropriate. Furthermore, circ-BOK-6 expression status was not shown to be associated with mitotic rate (Ki-67 index), HER2, ER, or PR status, as shown by the chi-square (χ^2^) test.

### 3.3. Low circ-BOK-6 Expression Predicts Poor Prognosis in BC Patients, Independently of Other Clinicopathological Factors

**Figure 3:**
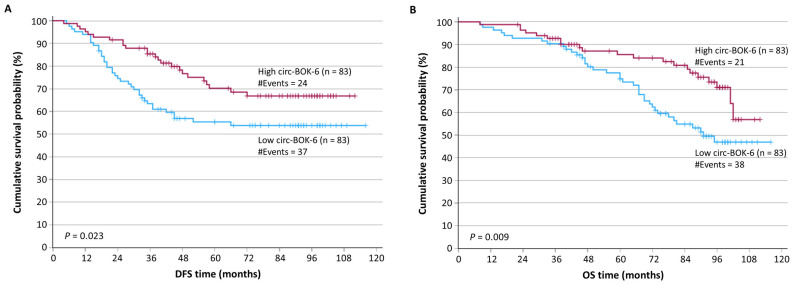
Kaplan–Meier survival analysis for the DFS (**A**) and OS (**B**) of BC patients, based on circ-BOK-6 expression status. Lower expression of circ-BOK-6 was an unfavorable prognosticator. The *p*-values were calculated by using the Mantel–Cox (log-rank) test.

After splitting at the median value to classify circ-BOK-6 expression in each tissue specimen as positive or negative, survival analysis was performed. This included Kaplan–Meier analysis and Cox regression, examining correlations with both the DFS and OS. Particularly in Cox regression, bootstrapping based on 1000 samples was applied to strengthen any conclusions. Follow-up data was available for 166 BC patients; the median follow-up time was 95 months. Kaplan–Meier survival curves depicted that circ-BOK-6–negative BC patients were more likely to present with a relapse and succumb to their disease, compared to patients with circ-BOK-6–negative breast tumors (*p* = 0.023 and *p* = 0.009, respectively) (Figure 3A,B).

In accordance with Kaplan–Meier survival analysis results, univariate Cox regression analysis showed that BC patients with circ-BOK-6–positive expression status had a 2-fold lower risk of tumor recurrence, in comparison with circ-BOK-6–negative ones (HR = 0.56, 95% CI = 0.33–0.93, *p* = 0.025). Besides that, the molecular subtype and prognostic stage of the disease were also significant indicators of prognosis with regard to DFS (*p* < 0.001 for both these variables). Therefore, the multivariate Cox regression analysis included the circ-BOK-6 expression status and the prognostic stage (Table 3), or alternatively, the circ-BOK-6 expression status, anatomic stage, and molecular subtype (Table S2). Interestingly, even after performing bootstrapping, patients with breast tumors expressing circ-BOK-6 expression status were less likely to relapse (Table S3). The prognostic significance of circ-BOK-6 expression status was independent of any other established prognosticator examined in the current study.

Furthermore, univariate Cox regression analysis showed that BC patients with circ-BOK-6–positive expression status had a 2-fold lower risk of dying from BC, in comparison with circ-BOK-6–negative ones (HR = 0.50, 95% CI = 0.29–0.85, *p* = 0.011). Besides that, the molecular subtype and prognostic stage of the disease were also significant prognostic factors of OS (*p* < 0.001 and *p* = 0.001, respectively). Thus, the multivariate Cox regression analysis included the circ-BOK-6 expression status and the prognostic stage (Table 4), or alternatively, the circ-BOK-6 expression status, anatomic stage, and molecular subtype (Table S4). Interestingly, even after performing bootstrapping, BC patients bearing circ-BOK-6–positive tumors had a significantly shorter OS time interval than those with inferior circ-BOK-6 levels, independently of any other established predictor of OS (Table S5).

### 3.4. Prognostic Value of circ-BOK-6 Expression in BC Patients, Stratified According to Molecular Subtype, Anatomic Stage, or Prognostic Stage

The molecular subtype of the tumor, as well as the anatomic and prognostic stages, are critical factors for the prognosis of BC patients. For this reason, we stratified patients according to these variables so as to further assess the potential additional impact of circ-BOK-6 expression status in each subgroup.

**Figure 4:**
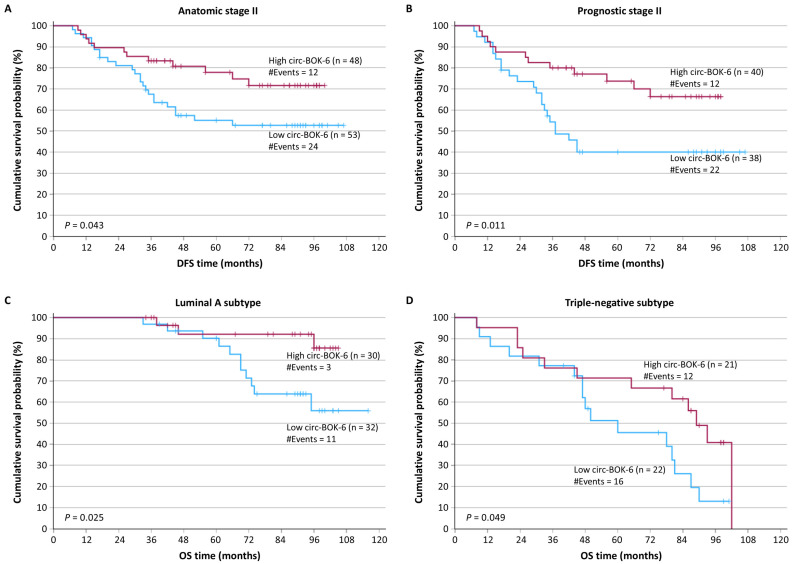
Stratified Kaplan–Meier survival curves for the DFS and OS of BC patients, according to anatomic stage, prognostic stage, and molecular subtype of tumors. Lower expression of circ-BOK-6 was an unfavorable prognosticator for DFS, particularly among BC patients of anatomic stage II (**A**) or prognostic stage II (**B**). Moreover, low circ-BOK-6 expression predicted poorer OS, particularly among BC patients with tumors of luminal A (**C**) or TNBC subtype (**D**). The *p*-values were calculated by using the Mantel–Cox (log-rank) test.

As illustrated in Kaplan–Meier DFS curves (Figure 4A,B), patients of anatomic stage II or prognostic stage II with circ-BOK-6–negative breast tumors had lower survival rates (*p* = 0.043 and *p* = 0.011, respectively), in comparison with those with circ-BOK-6–negative tumors of the same anatomic stage or prognostic stage.

Furthermore, BC patients with luminal A subtype and low circ-BOK-6 expression showed an increased probability of succumbing to their disease as compared to those bearing circ-BOK-6–negative tumors of luminal A molecular subtype (*p* = 0.025) (Figure 4C). Similarly, yet marginally, as depicted in Figure 4D, TNBC patients negative for circ-BOK-6 expression had poorer OS, as compared to those bearing circ-BOK-6–negative triple-negative tumors (*p* = 0.049).

## 4. Discussion

In this study, we demonstrated that circ-BOK-6 is lower in BC and serves as an independent prognostic indicator for recurrence and mortality. Inferior intracellular levels of circ-BOK-6 were noticed in the majority of breast tumors compared to matched non-cancerous breast tissues. The limited number of paired samples is a limitation of our study. This, together with the overlap in expression distributions between all malignant and normal tissues, suggests limited diagnostic utility of this circRNA in BC, if any. Nonetheless, the observed alteration in circ-BOK-6 expression during breast tumorigenesis warrants further investigation. Although several studies have reported a global reduction in circRNAs in cancer, this concept remains under debate in the context of BC [23,24]. An alternative explanation is that circRNA expression patterns largely reflect dysregulation of their parental genes [25,26]. Given that BOK is a protein facilitating apoptosis, its reduced mRNA and protein expression in BC may represent a key adaptive mechanism by which malignant cells evade apoptosis. Moreover, low BOK expression in BC cells promotes epithelial–mesenchymal transition and cell migration via the Wnt signaling pathway [19]. In this context, suppression of the host gene may account for the decreased circ-BOK-6 levels observed in tumor tissues relative to adjacent normal counterparts, potentially reflecting an early event in breast carcinogenesis.

Low expression levels of this circRNA within the patient cohort were strongly associated with poor clinical outcomes. Notably, both Kaplan–Meier survival analysis and Cox regression modeling demonstrated that negative circ-BOK-6 expression was associated with a 2-fold higher risk of tumor recurrence and disease-specific mortality, independently of established prognostic factors. These findings suggest that, in a subset of breast tumors, circ-BOK-6 may exert a tumor suppressor function, in accordance with the tumor-suppressive role of its parental *BOK* gene [14,27,28]. In fact, BOK has been shown to display cell death-independent tumor suppressor activity in cancer. Interestingly, BOK seems to be involved in the regulation of a variety of other, “apoptosis-independent” cellular functions, including the unfolded protein response, cellular proliferation, metabolism, and autophagy [29,30]. Moreover, many circRNAs derived from apoptosis-related genes are increasingly recognized as components of complex RNA regulatory networks, with emerging evidence supporting their tumor suppressor potential through sequestration of specific miRNAs [20,31]; particularly, circ-BOK-6 is very likely to bind miR-4267 [20], the role of which has not been studied yet. In general, miRNA-sponging activity of circRNAs affects gene expression at the post-transcriptional level [32,33].

Besides their miRNA-sponging activity, circRNAs localized in the nucleus can activate host gene transcription through recruitment of RNA polymerase II and U1 small nuclear ribonucleoprotein [34]. Given that the localization of circ-BOK-6 is currently unknown, such a way of action could not be excluded. circ-BOK-6 is also very likely to possess three m^6^A sites [20], which—along with the open reading frame it possesses—may imply a protein-coding role for this circRNA. Future studies should explore the contribution of RNA modifications and internal ribosome entry sites (IRES) to circRNA function. Bridging the gap between experimental findings and clinical application will require a deeper understanding of circRNA biogenesis, intracellular trafficking, and translational potential, including the production of small peptides and their impact on the post-translational regulation of parental genes [35,36].

Importantly, circ-BOK-6 expression retains its prognostic significance across distinct molecular and clinical subgroups of patients. In particular, inferior circ-BOK-6 levels were associated with significantly poorer OS in patients with luminal A BC or TNBC. Luminal A BC patients typically respond well to endocrine therapies, such as tamoxifen and aromatase inhibitors, and are less frequently treated with chemotherapy [37,38]. Consequently, molecular profiling is often employed to refine relapse risk assessment and guide decisions regarding adjuvant chemotherapy [37]. In this context, circ-BOK-6 expression may represent a valuable biomarker for identifying high-risk luminal A patients who could benefit from closer monitoring or more aggressive therapeutic strategies. Similarly, the assessment of circ-BOK-6 expression may improve risk stratification in TNBC patients. This is particularly relevant in light of the well-described “TNBC paradox,” whereby patients often exhibit high initial response rates to neoadjuvant chemotherapy, yet do not achieve corresponding improvements in OS, due to an elevated risk of early relapse and metastatic progression [39,40]. Furthermore, negative circ-BOK-6 status enables more precise prognostic discrimination among patients with both anatomic and prognostic stage II disease, identifying individuals with a substantially higher likelihood of recurrence within these heterogeneous groups. Collectively, these findings suggest that low circ-BOK-6 expression could serve as a complementary biomarker alongside established stage classification systems and molecular subtyping, thereby enhancing the identification of patients at increased risk of disease recurrence [41]. Moreover, loss of circ-BOK expression could be exploited in a multiparametric model based on molecular signatures [42].

Although our findings highlight the significant potential of circ-BOK-6 as a prognostic biomarker in BC, several limitations of the present study should be considered. Primarily, a fundamental limitation is the limited number of available control samples (only 44), which restricted a comprehensive assessment of its diagnostic discriminatory capacity. Moreover, the retrospective, single-center design, along with the moderate cohort size, may limit the generalizability of the results. Consequently, validation in independent, larger, multi-center cohorts is indispensable to confirm the clinical utility of circ-BOK-6.

In addition, despite the robustness of the clinical associations observed, the precise biological function of circ-BOK-6 remains to be elucidated. Importantly, it should be noted that our study aimed only at a preliminary examination of the potential clinical significance of circ-BOK-6 as a molecular tissue biomarker in BC. Thus, every prediction regarding putative m^6^A sites of circ-BOK-6 and potentially sponged miRNAs was previously performed using publicly available algorithms [20], yet no such regulatory element has been validated so far. To bridge this translational gap, future functional studies are necessary to validate such regulatory RNA elements of circ-BOK-6 and clarify the mechanisms underlying its altered expression during breast carcinogenesis, as well as to explore its potential roles in apoptosis, regulation of its parental gene, and RNA-protein interactions. Moreover, massive parallel circRNA sequencing could reveal several other circular transcripts exhibiting significant differential expression among distinct prognostic groups of female BC patients.

## 5. Conclusions

In conclusion, low circ-BOK-6 expression was associated with increased risk of tumor recurrence and reduced OS in female BC patients. Notably, low circ-BOK-6 expression may serve as an independent prognostic indicator, providing additional clinical value beyond established clinicopathological parameters, including molecular subtype and disease stage. The integration of circ-BOK-6 expression with existing prognostic factors may further enhance risk stratification of BC patients across different molecular subtypes.

## Figures and Tables

**Table 1:** Breast tumor staging and molecular subtype results.

	Number of Patients (%)
**Anatomic stage**	
I	42 (24.4%)
II	106 (61.6%)
III	24 (14.0%)
**Molecular subtype**	
Luminal A	66 (38.4%)
Luminal B	43 (25.0%)
Triple-negative	44 (25.6%)
HER2-enriched	19 (11.0%)
**Prognostic stage**	
I	61 (35.5%)
II	82 (47.7%)
III	29 (16.8%)

**Table 2:** circ-BOK-6 levels in cancerous and non-cancerous breast tissue specimens.

Variable	Mean ± SE	Range	Percentiles		
			25th	50th (Median)	75th
circ-BOK-6 expression (RQU)					
in cancerous tissues (*n* = 172)	85.60 ± 14.82	0.11–691.05	0.52	14.82	111.37
in non-cancerous tissues (*n* = 44)	49.54 ± 20.57	0.84–205.71	4.66	20.57	85.12

Abbreviations: RQU, relative quantification units; SE, standard error.

**Table 3:** Univariate and multivariate Cox regression models for BC patients’ DFS prediction.

	Univariate Analysis (*n* = 166)			Multivariable Analysis (*n* = 166)		
Covariate	HR	95% CI	*p* Value ^1^	HR	95% CI	*p* Value ^1^
circ-BOK-6 expression status						
Negative (*n* = 83)	1.00			1.00		
Positive (*n* = 83)	0.56	0.33–0.93	*0.025*	0.51	0.31–0.86	*0.011*
Prognostic stage			*<0.001*			*<0.001*
I (*n* = 60)	1.00			1.00		
II (*n* = 78)	3.29	1.62–6.66	*0.001*	3.48	1.71–7.06	*0.001*
III (*n* = 28)	5.48	2.50–12.01	*<0.001*	5.73	2.61–12.59	*<0.001*

^1^ Statistically significant *p* values are shown in italics. Abbreviations: CI, confidence interval; HR, hazard ratio.

**Table 4:** Univariate and multivariate Cox regression models predicting the OS of BC patients.

	Univariate Analysis (*n* = 166)			Multivariable Analysis (*n* = 166)		
Covariate	HR	95% CI	*p* Value ^1^	HR	95% CI	*p* Value ^1^
circ-BOK-6 expression status						
Negative (*n* = 83)	1.00			1.00		
Positive (*n* = 83)	0.50	0.29–0.85	*0.011*	0.49	0.28–0.83	*0.008*
Prognostic stage			*0.001*			*0.001*
I (*n* = 60)	1.00			1.00		
II (*n* = 78)	2.93	1.47–5.82	*0.002*	2.96	1.49–5.87	*0.002*
III (*n* = 28)	4.30	1.96–9.44	*<0.001*	4.38	2.00–9.59	*<0.001*

^1^ Statistically significant *p* values are shown in italics. Abbreviations: CI, confidence interval; HR, hazard ratio.

## Data Availability

The original contributions presented in this study are included in the article and Supplementary Material. Further inquiries can be directed to the corresponding author.

## Supplementary Materials

The following supporting information can be downloaded at: https://www.mdpi.com/article/10.3390/biomedicines14051118/s1. Table S1: Biological features of malignant breast tumors; Table S2: Univariate and alternative multivariate Cox regression analyses for BC patients’ DFS prediction; Table S3: Bootstrapped univariate and multivariate Cox regression analyses for BC patients’ DFS prediction; Table S4: Univariate and alternative multivariate Cox regression analyses for BC patients’ OS prediction; Table S5: Bootstrapped univariate and multivariate Cox regression analyses for BC patients’ OS prediction.
