# Supplementary material for: Low Expression of a Circular Transcript of the Apoptosis Regulator Gene BOK Is Associated with Unfavorable Prognosis in Breast Cancer
Source: Biomedicines. 2026 May 15;14(5):1118. doi: 10.3390/biomedicines14051118 (PMC13204483; doi:10.3390/biomedicines14051118)
Supplement: Supplementary file 1 [file biomedicines-14-01118-s001.zip › Table S1.pdf]

Table S1. Biological features of malignant breast tumors.

	Number of patients (%)
**Patients**	172
**Age (y)**	Median: 59; Range: 32 – 90
**Tumor size (cm^2)**	Median: 2.4; Range: (0.6 – 8.5)
**Cancer type**	
Invasive ductal carcinoma	137 (79.7%)
Lobular carcinoma	15 (8.7%)
Other carcinomas	20 (11.6%)
**Histological grade**	
I	7 (4.1%)
II	113 (65.7%)
III	52 (30.2%)
**HER2 status**	
Negative	132 (79.0%)
Positive	35 (21.0%)
Unknown	5
**ER status**	
Negative	70 (40.7%)
Positive	102 (59.3%)
**PR status**	
Negative	96 (55.8%)
Positive	76 (44.2%)
**Ki-67 index***	
Low (≤14%)	107 (64.5%)
High (>14%)	59 (35.5%)
Unknown	6

Abbreviations: ER, estrogen receptor; PR, progesterone receptor.
