# Supplementary material for: Low Expression of a Circular Transcript of the Apoptosis Regulator Gene BOK Is Associated with Unfavorable Prognosis in Breast Cancer
Source: Biomedicines. 2026 May 15;14(5):1118. doi: 10.3390/biomedicines14051118 (PMC13204483; doi:10.3390/biomedicines14051118)
Supplement: Supplementary file 1 [file biomedicines-14-01118-s001.zip › Table S2.pdf]

Table S2. Univariate and alternative multivariate Cox regression analyses for BC patients' DFS prediction.

	Univariate Analysis (*n* = 166)			Multivariable Analysis (*n* = 166)		
**Covariate**	**HR**	**95% CI**	***P* value [1]**	**HR**	**95% CI**	***P* value [1]**
circ-BOK-6 expression status						
Negative (*n*=83)	1.00			1.00		
Positive (*n*=83)	0.56	0.33 – 0.93	*0.025*	0.43	0.25 – 0.73	*0.002*
Anatomic stage			*0.002*			*0.034*
I (*n*=42)	1.00			1.00		
II (*n*=101)	1.62	0.80 – 3.27	0.18	1.14	0.55 – 2.35	0.72
III (*n*=23)	3.87	1.73 – 8.63	*0.001*	2.53	1.07 – 6.01	*0.035*
Molecular subtype			*<0.001*			*0.001*
Luminal A (*n*=62)	1.00			1.00		
Luminal B (*n*=42)	1.06	0.47 – 2.35	0.89	1.24	0.55 – 2.79	0.61
Triple-negative (*n*=43)	3.31	1.73 – 6.31	*<0.001*	3.20	1.64 – 6.25	*0.001*
HER2-enriched (*n*=19)	3.72	1.73 – 8.00	*0.001*	3.93	1.78 – 8.68	*0.001*

[1] Statistically significant *P* values are shown in italics.
Abbreviations: CI, confidence interval; HR, hazard ratio.
