# Supplementary material for: Low Expression of a Circular Transcript of the Apoptosis Regulator Gene BOK Is Associated with Unfavorable Prognosis in Breast Cancer
Source: Biomedicines. 2026 May 15;14(5):1118. doi: 10.3390/biomedicines14051118 (PMC13204483; doi:10.3390/biomedicines14051118)
Supplement: Supplementary file 1 [file biomedicines-14-01118-s001.zip › Table S3.pdf]

Table S3. Bootstrapped univariate and multivariate Cox regression analyses for BC patients' DFS prediction.

Covariate	Univariate Analysis (*n* = 166)			Multivariable Analysis (*n* = 166)					
	**HR**	**BCa bootstrap 95% CI**	**Bootstrap *P* value [1]**	**HR**	**BCa bootstrap 95% CI**	**Bootstrap *P* value [1]**	**HR**	**BCa bootstrap 95% CI**	**Bootstrap *P* value [1]**
circ-BOK-6 expression status									
Negative (*n*=83)	1.00			1.00			1.00		
Positive (*n*=83)	0.56	0.34 – 0.86	*0.018*	0.51	0.30 – 0.84	*0.016*	0.43	0.23 – 0.68	*0.003*
Anatomic stage									
I (*n*=42)	1.00						1.00		
II (*n*=101)	1.62	0.82 – 4.11	0.17				1.14	0.54 – 2.86	0.73
III (*n*=23)	3.87	1.72 – 10.85	*0.001*				2.53	1.08 – 7.11	*0.033*
Molecular subtype									
Luminal A (*n*=62)	1.00						1.00		
Luminal B (*n*=42)	1.06	0.43 – 2.53	0.89				1.24	0.50 – 2.97	0.61
Triple-negative (*n*=43)	3.31	1.69 – 7.44	*0.001*				3.20	1.59 – 7.43	*0.001*
HER2-enriched (*n*=19)	3.72	1.76 – 8.62	*0.001*				3.93	1.57 – 9.59	*0.001*
Prognostic stage									
I (*n*=60)	1.00			1.00					
II (*n*=78)	3.29	1.71 – 9.33	*0.002*	3.48	1.72 – 9.94	*0.002*			
III (*n*=28)	5.48	2.50 – 15.58	*0.001*	5.73	2.50 – 18.13	*0.001*			

[1] Statistically significant *P* values are shown in italics.

Abbreviations: BCa, bias-corrected and accelerated; CI, confidence interval; HR, hazard ratio.
