# Supplementary material for: Low Expression of a Circular Transcript of the Apoptosis Regulator Gene BOK Is Associated with Unfavorable Prognosis in Breast Cancer
Source: Biomedicines. 2026 May 15;14(5):1118. doi: 10.3390/biomedicines14051118 (PMC13204483; doi:10.3390/biomedicines14051118)
Supplement: Supplementary file 1 [file biomedicines-14-01118-s001.zip › Table S4.pdf]

Table S4. Univariate and alternative multivariate Cox regression analyses for BC patients' OS prediction.

	**Univariate Analysis (*n* = 166)**			**Multivariable Analysis (*n* = 166)**		
**Covariate**	**HR**	**95% CI**	***P* value** [1]	**HR**	**95% CI**	***P* value** [1]
circ-BOK-6 expression status						
Negative (*n*=83)	1.00			1.00		
Positive (*n*=83)	0.50	0.29 – 0.85	*0.011*	0.45	0.26 - 0.77	*0.003*
Anatomic stage			0.065			0.79
I (*n*=42)	1.00			1.00		
II (*n*=101)	1.82	0.90 – 3.67	0.097	1.21	0.59 - 2.51	0.60
III (*n*=23)	2.72	1.17 – 6.31	*0.020*	1.38	0.56 - 3.41	0.49
Molecular subtype			*<0.001*			*<0.001*
Luminal A (*n*=62)	1.00			1.00		
Luminal B (*n*=42)	0.94	0.41 – 2.18	0.89	0.98	0.42 - 2.27	0.96
Triple-negative (*n*=43)	4.01	2.10 – 7.64	*<0.001*	4.05	2.07 - 7.94	*<0.001*
HER2-enriched (*n*=19)	2.44	1.02 – 5.84	*0.045*	2.44	1.01 - 5.85	*0.046*

[1] Statistically significant *P* values are shown in italics.
Abbreviations: CI, confidence interval; HR, hazard ratio.
