# Supplementary material for: Low Expression of a Circular Transcript of the Apoptosis Regulator Gene BOK Is Associated with Unfavorable Prognosis in Breast Cancer
Source: Biomedicines. 2026 May 15;14(5):1118. doi: 10.3390/biomedicines14051118 (PMC13204483; doi:10.3390/biomedicines14051118)
Supplement: Supplementary file 1 [file biomedicines-14-01118-s001.zip › Table S5.pdf]

Table S5. Bootstrapped univariate and multivariate Cox regression analyses for BC patients' OS prediction.

	Univariate Analysis (*n* = 166)			Multivariable Analysis (*n* = 166)					
**Covariate**	**HR**	**BCa bootstrap 95% CI**	**Bootstrap *P* value [1]**	**HR**	**BCa bootstrap 95% CI**	**Bootstrap *P* value [1]**	**HR**	**BCa bootstrap 95% CI**	**Bootstrap *P* value [1]**
circ-BOK-6 expression status									
Negative (*n*=83)	1.00			1.00			1.00		
Positive (*n*=83)	0.50	0.29 – 0.81	*0.011*	0.49	0.27 – 0.80	*0.008*	0.45	0.27 – 0.67	*0.006*
Anatomic stage									
I (*n*=42)	1.00						1.00		
II (*n*=101)	1.82	0.89 – 4.87	0.080				1.21	0.55 – 2.93	0.60
III (*n*=23)	2.72	1.01 – 7.93	*0.020*				1.38	0.47 – 4.21	0.49
Molecular subtype									
Luminal A (*n*=62)	1.00						1.00		
Luminal B (*n*=42)	0.94	0.40 – 2.14	0.89				0.98	0.36 – 2.31	0.97
Triple-negative (*n*=43)	4.01	2.25 – 7.96	*0.001*				4.05	1.93 – 9.71	*0.001*
HER2-enriched (*n*=19)	2.44	0.89 – 5.45	*0.033*				2.44	0.79 – 7.45	0.071
Prognostic stage									
I (*n*=60)	1.00			1.00					
II (*n*=78)	2.93	1.50 – 6.29	*0.003*	2.96	1.50 – 7.29	*0.002*			
III (*n*=28)	4.30	1.88 – 10.98	*0.001*	4.38	1.94 – 12.56	*0.001*			

[1] Statistically significant *P* values are shown in italics.

Abbreviations: BCa, bias-corrected and accelerated; CI, confidence interval; HR, hazard ratio.
